# Managed Long-Term Care Services: A Playbook Innovation or a Hail Mary?

**DOI:** 10.1093/geroni/igab046.1748

**Published:** 2021-12-17

**Authors:** Larry Polivka, Robert Applebaum

## Abstract

The approach to providing long-term services and supports (LTSS) has changed dramatically over the last three decades in both the financing and delivery arenas. In the U.S., long-term strategies have varied by state in organizational structure, scope of delivery and administrative practices. In the past two decades an additional change has emerged with over half the states adopting some form of managed LTSS. This shift has deepened the divide in state approaches to LTSS system design and delivery. The shift to managed LTSS has been largely fueled by ideological expectations and concerns about growing Medicaid costs: Empirical research findings have played a minimal role. For example, the large CMS evaluation conducted in this area did not include Medicaid data or encounter data from the managed care plans as part of the study efforts. However, the managed LTSS experiment does create an opportunity to compare costs and outcomes of these different models of financing and delivery. This symposium will present preliminary evaluation findings from two states, Ohio and Pennsylvania, which are generating data to assess both the implementation and outcomes of these alternative LTSS models. To set the context an initial paper will discuss the expansion of managed LTSS programs across the nation and examine how these efforts compare to the development occurring in the European LTSS systems. The third presentation will discuss the results of the Community Catalyst dual eligibles’ managed care demonstration program monitoring project.

